# Early Levodopa-Induced Motor Complications in RAB39B X-Linked Parkinsonism

**DOI:** 10.5334/tohm.946

**Published:** 2024-11-25

**Authors:** Laurane Mackels, David Aktan, Frédérique Depierreux

**Affiliations:** 1 Neurology Department, Regional Hospital of Liege, Liege, Belgium; 2 Neurology Department, University Hospital of Liege, Liege, Belgium; 3 GIGA –CRC *in vivo*imaging, Rare Movement Disorders Research Group, University of Liege, Liege, Belgium

**Keywords:** Levodopa-induced motor complication, monogenic parkinsonism, RAB39B, synucleinopathy

## Abstract

**Background:**

While levodopa may benefit some patients with monogenic Parkinson’s Disease and parkinsonism, others may exhibit aberrant responses earlier after exposure. Reporting treatment responses in rare genetic parkinsonism will help tailor therapeutic approaches to specific patients subpopulations.

**Case Report:**

We report the therapeutic response in a patient with *RAB39B* X-linked parkinsonism, who exhibited motor and non-motor complications within a few months of Levodopa.

**Discussion:**

Severe and debilitating Levodopa-induced complications can occur very early in the treatment course of X-linked parkinsonism, highlighting the need for an individualized therapeutic approach and follow-up in rare parkinsonian syndromes.

## 1. Introduction

Typical Parkinson’s Disease (PD) is characterized by a good initial response to levodopa treatment [1], with treatment-related complications appearing later in the disease progression, usually years afterward [2]. While some patients with Early-Onset Parkinson’s Disease (EOPD) and Early Onset parkinsonism (EO parkinsonism) may similarly benefit from this treatment [3], motor complication may arise earlier after drug exposure [4,5]. Reporting unsual therapeutic responses in rare genetic PD and parkinsonism will help tailor therapeutic approaches to specific patients subpopulations. In this report, we aim to comprehensively illustrate the range of early motor complications experienced by our patient presenting with *RAB39B* X-linked parkinsonism, within the first year following treatment with levodopa. This holds importance considering the notably brief duration of levodopa exposure, contrasting with the typical timeframes reported for these complications. Acquiring such information in rare conditions is challenging given the scarcity of patients. Our objective is to share documentation of therapeutic responses within this specific population, ultimately aiming to refine and optimize treatment strategies.

## 2. Case Description

**Figure 1:**
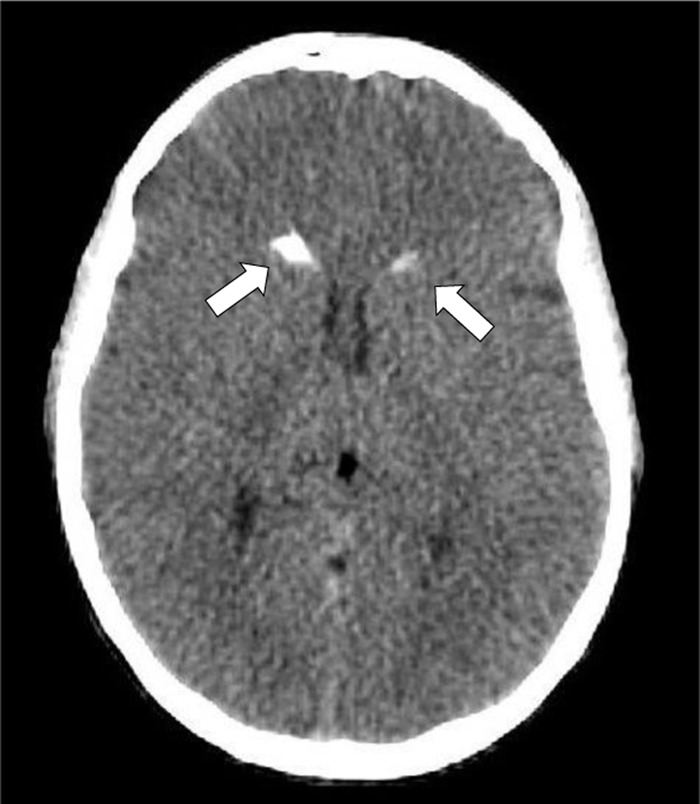
Brain window of low-dose head CT showing bilateral calcification in CN (white arrows). Figure adapted from a previous article [6].

We previously reported the clinical presentation and imaging of a 37-year-old man harbouring a *de novo* substitution mutation (c.216–2A>G) in *RAB39B* gene, known to cause X-linked PD and parkinsonism [6]. Our patient presented with childhood-onset axial hypotonia at the age of 11 months, with macrocephalia noted at 2 years old (>95^th^ percentile). He presented with neurodevelopmental delays, autistic traits, and had a generalized tonic-clonic seizure in adolescence. Neuropsychological assessment showed impaired short-term memory, attention, verbal fluency and executive functions. We described multimodal imaging acquired at age 37 [6]. Notably, our patient displayed vermian hypoplasia, shortened corpus callosum and asymmetrical calcifications of caudate nuclei on brain computed tomography (Figure 1) [6]. T2*W-GRE sequences showed hypointense signals in both the globus pallidus and substantia nigra (SN), consistent with deposits of para- or diamagnetic substances [6]. Transcranial ultrasound highlighted iron accumulation in the *pars compacta* of the SN. [^18^F]FDG PET indicated cortical hypometabolism in the frontal, supra-orbital and temporal regions, as well as in the thalami and left striatum [6]. DAT-scan showed bilateral posterior putamen hypoactivity, suggesting significant dysfunction in presynaptic dopaminergic pathways [6].

Considering the severe and rapidly worsening parkinsonism (video appendix 1A-B), we started treatment at the age of 38 with half a Prolopa 250 mg tablet three times daily and one Prolopa HBS 125 mg tablet at night, initially yielding a positive motor response (video appendix 2A-B). However, levodopa-induced motor complications emerged progressively after a few months, peaking in severity approximately 10 months later following incremental increases to a maximum daily regimen of five Prolopa tablets, one Xadago 100 mg tablet, and one HBS 125 mg tablet at night. The patient exhibited the whole range of complications, including wearing-OFF, delayed-ON and ON-OFF phenomenon, freezing and dyskinesias (video appendix 3A-C). He also developed auditory hallucinations and dopamine dysregulation syndrome. Complementary medications including safinamide and clozapine were subsequently introduced, without significant improvement.

**Video 1:** Illustration of parkinsonian syndrome, response to levodopa and early motor complications in a patient with RAB39B X-linked Parkinson Disease. Part 1: Parkinsonian syndrome before treatment. This video highlights features of the parkinsonian syndrome, before starting treatment with levodopa. **(A)** Hypokinetic decrement occurs when the patient performs the finger-to-nose test. **(B)** The patient’s gait is characterized by rigidity, slowing of gait, reduced arm swing and shorter step length. Part 2: Response to treatment. This video illustrates the clinical improvement following treatment with levodopa. **(A)** Steps lengths and walking speed are increased. **(B)** Bradykinesia and hypokinetic decrement are less pronounced when compared to video 1. Part 3: Early-motor complications. This video demonstrates early-motor complications that appeared less than one year after levodopa initiation, including dyskinesias affecting **(A)** the trunk, the lower limbs, **(B)** and the gait. **(C)** Motor fluctuations are also illustrated with a freezing phenomenon.

## 3.Discussion

EO parkinsonism differs from idiopathic PD (iPD) in terms of genetics and clinical aspects. EO parkinsonism is much more frequently caused by monogenic mutations than iPD and manifests with an earlier age of onset, before age 40 to 50. Parkinsonian features may present within more complex syndromes, alongside other neurological signs like dystonia, pyramidal abnormalities, cognitive decline, as well as psychiatric symptoms, systemic issues, and brain imaging alterations [4,7]. EO parkinsonism may greatly benefit from levodopa therapy, such as those with recessively inherited *PRKN*-associated PD or dominantly inherited *LRKK2*-associated PD [7].

Although the mechanisms underlying this susceptibility remain unknown, some patients with EO parkinsonism appear prone to developing motor complications sooner in the disease’s course or after briefer levodopa exposure [4]. As such, early levodopa complications have been documented in several monogenic EO parkinsonism and juvenile parkinsonism (JOPD), including *SNCA, GBA, DNAJC6* and *FBXO7*-related PD [3]. Of the 20 genes causing Mendelian forms of PD, *RAB39B* is the only one with X-linked inheritance. Affected males present with early-onset parkinsonism, intellectual disability, macrocephaly, and sometimes seizures [8]. We previously reported the first multimodal imaging data in one male patient and summarized clinical and imaging evidence available in published patients to date [6]. *Post-mortem* analysis in one patient have highlighted pathological feature of alpha-synucleopathy [8]. With only a few patients described in the literature, data regarding response to levodopa in this specific population are sparse (Table 1) but suggest that some patients may respond to treatment. Although levodopa-induced dystonia has been reported, motor complications were described in one patient only, 8 years after treatment initiation (Table 1).

**Table 1:** Summary of data regarding response to levodopa in patients with RAB39B X-linked Parkinsonism.


REF.	MUTATION	SEX	AGE AT SYMPTOM’S ONSET	RESPONSE TO L-DOPA^1^	LDIC²	DELAY TO LDIC	DBS

[19,20]	Point mutation c.503C>A [p.Thr168Lys]	13M and 1F	10–20’s	None (3/3)	Yes	NR	NR

[8]	All gene deletion 45kb	M	Late childhood	NA	NA	NA	NR

[8]	All gene deletion 45kb	M	38y	Yes	No	NA	NR

[8]	All gene deletion 45kb	M	44y	Yes	Yes	NR	NR

[21]	Nonsense mutation c.557G.Ain exon 2 [p.Trp186stop]	M	39y	Good	Yes (MF, dyskinesia, and limb dystonia)	8 years	Not performed

[22]	Missense mutation (c.574G > A; p.G192R)	5M	29y–31y–48y–50y–53y	Yes (4/4)	Yes (3/4 – dyskinesia)	NR	NR

[22]	Missense mutation (c.574G > A; p.G192R)	2F	55y–57y	Yes (2/2)	Yes (1/2 dyskinesia)	NR	NR

[23]	Frameshift mutation c. 536dupA (p.E179fsX48)	M	10y	Partial	NR	NA	NR

[23]	Frameshift mutation c. 536dupA (p.E179fsX48)	M	12y	None	NR	NA	NR

[24]	Frameshift variant c.137dupT	M	11y	Good	No	NA	NR

[24]	Frameshift variant c.137dupT	M	60y	Good	Yes (dyskinesia)	NR	NR

[24]	Deletion variant c.371delA	M	44y	Good	No	NA	NR

[25]	Hemizygous single base pair deletion c.432delA	M	29y	Partial	NR	NA	NR


^1^ Number of patients bracketed. ^2^ Levodopa induced complications with number of patients and type of complication bracketed. F: Female; LDIC: Levodopa induced complications; M: Male; MF: Motor Fluctuations; NA: Not applicable; NR: Not reported.

Our patient and caregivers reported significant improvement following treatment initiation, confirmed by improved UPDRS scores. However, unlike typical alpha-synucleinopathy, motor complications emerged early, within a few months of treatment initiation. These motor complications were rapidly severe, significantly impairing patient’s quality of life and manifesting as prominent ON-OFF phenomenon, freezing and dyskinesia, similar to those observed after several years of treatment in classical PD. Reporting these exceptionally early and severe levodopa-induced complications provides novel and valuable insights into the therapeutic response in this rare population.

The duration of levodopa therapy was long considered an important driver of levodopa-induced complications [9,10]. However, recent studies suggest that treatment complications like motor fluctuations and dyskinesias in iPD may instead reflect longer disease duration and higher daily levodopa doses [11]. Consistent with these findings, higher rates of motor complications have been observed in patients with monogenic EOPD [12,13,14,15], where neurodegeneration may occur over many years before treatment initiation. Early occurrence of motor complication in our patient align with this observation. Conversely, some patients with pathogenic *RAB39b* mutation exhibited only mild or no levodopa-induced motor complication (Table 1). Moreover, complications do not develop in all individuals with EOPD [12]. This indicates that other factors are significant in the development of motor complications.

Given our patient’s severe phenotype and the need for adjunctive treatments to manage motor complications, further therapeutic options must be investigated. Based on expert discussions within the local reference centre and reviews of evidence across the literature, deep brain stimulation (DBS) is strongly considered. DBS has shown promise in monogenic PD and parkinsonism patients like *LRRK2* [16]. However, it’s vital to consider potential complications that may occur upon DBS implantation, including cognitive decline and dyskinesias such as seen in *SNCA, GBA* and *PRKN*-PD [17,18].

In summary, we reported multiple levodopa-induced motor complications occurring within first year of treatment in a male patient with X-linked parkinsonism due to *RAB39B* pathogenic variants. Neurologists should be aware that an initially positive response to treatment can quickly lead to devastating motor complications, justifying close monitoring of the patient, in contrast to the slower progression seen in classical alpha-synucleinopathies, such as iPD. Given the complexity and rarity of this condition, the optimal therapeutic approach remains unknown. Collaborative data collection is crucial to pave the way for future therapeutic guidance and developments in these rare conditions.
